# The Segregation of p.Arg68Ter-*CLDN14* Mutation in a Syrian Deaf Family, Phenotypic Variations, and Comparative Analysis with the *GJB2* Gene

**DOI:** 10.3390/genes15050588

**Published:** 2024-05-06

**Authors:** Abdelaziz Tlili, Abdullah Al Mutery, Jihen Chouchen

**Affiliations:** 1 Department of Applied Biology, College of Sciences, University of Sharjah, Sharjah 27272, United Arab Emirates; 2 Human Genetics and Stem Cell Laboratory, Research Institute of Sciences and Engineering, University of Sharjah, Sharjah 27272, United Arab Emirates; jihenchouchen@gmail.com

**Keywords:** *CLDN14*, non-syndromic autosomal hearing loss, p.Arg68Ter mutation, mutations review, genotype phenotype correlation, comparative analysis mutation rates *GJB2/CLDN14*

## Abstract

Hearing impairment, a rare inherited condition, is notably prevalent in populations with high rates of consanguinity. The most common form observed globally is autosomal recessive non-syndromic hearing loss. Despite its prevalence, this genetic disorder is characterized by a substantial genetic diversity, making diagnosis and screening challenging. The emergence of advanced next-generation sequencing (NGS) technologies has significantly advanced the discovery of genes and variants linked to various conditions, such as hearing loss. In this study, our objective was to identify the specific variant causing hearing loss in a family from Syria using clinical exome sequencing. The proband in the family exhibited profound deafness as shown by pure-tone audiometry results. The analysis of the different variants obtained by NGS revealed the presence of a nonsense mutation within the *CLDN14* gene. Through Sanger sequencing, we verified that this variant segregates with the disease and was not present in the control population. Moreover, we conducted a comprehensive review of all reported deafness-related *CLDN14* mutations and their associated phenotypes. Furthermore, we endeavored to carry out a comparative analysis between the *CLDN14* and *GJB2* genes, with the objective of identifying potential factors that could explain the notable discrepancy in mutation frequency between these two genes.

## 1. Introduction

Hearing loss (HL) is the most commonly occurring neurosensorial defect, affecting around 1/1000 children [1]. Genetic factors are responsible for a minimum of 60% of nonsyndromic sensorineural hearing loss cases [2]. Different inheritance patterns associated with this type of hearing loss were reported. Autosomal recessive nonsyndromic hereditary hearing loss (ARNSHL) is the most predominant form representing approximately 70% of all nonsyndromic hereditary hearing loss cases [1]. To date, mutations in over 75 genes have been identified as causes of ARNSHL “https://hereditaryhearingloss.org/ (accessed on 1 February 2024)”. Among these genes is *CLDN14*, which is associated with a specific form of ARNSHL known as DFNB29 [3].

*CLDN14*, that encodes for claudin proteins (~26 KDa), is involved in the regulation of epithelial barrier function, particularly at bicellular junctions [4]. This gene is expressed in various locations within the inner ear. The expression profile of *CLDN14* in the inner ear suggests its involvement in preserving the integrity of various compartments, most notably, the organ of Corti [5]. Murine studies showed a rapid deterioration of hair cells in the knockout *cldn14−/−* mouse, probably due to the basolateral parts of these cells being subjected to high levels of K+-rich endolymph, as a consequence of the absence/malfunction of Claudin 14 protein in the ion barrier [6].

So far, fourteen mutations in the *CLDN14* gene have been associated with ARSNHL [3,7,8,9,10,11,12,13,14,15,16,17]. The highest contribution and number of mutations have been reported in the Pakistani population, where the prevalence of *CLDN14* pathogenic variants was estimated to be 2.25% of deaf individuals [7]. It is worth noting that even though there is phenotypic variability associated with certain mutations, the impact of *CLDN14* mutations tends to be more severe at higher frequencies, with moderate to severe phenotypes reported at low frequencies, while severe to profound phenotypes often exhibited at higher frequencies [3,7,8,10,11,12,13,14].

Although the *CLDN14* gene is relatively small, it consists of a single coding exon, has a coding region of 720 nucleotides, and is associated with a limited number of mutations linked to hearing loss; another gene, *GJB2*, with a similar structure (one single coding exon) is also involved in deafness [18]. However, the *GJB2* gene exhibits a high number (approximately 135) of pathogenic variants associated with hearing loss [19]. This gene encodes the gap-junction connexin 26 (Cx26) protein, which serves as a subunit of gap junctions, facilitating communication between neighboring cells. Within the inner ear, Cx26 plays a crucial role in the maintenance of potassium homeostasis [20]. Within the spectrum of *GJB2* mutations, many variants have been reported as prevalent in specific populations. These include 35delG, 235delC, V37I, W24X, 167delT8, and R143W, which are common in Mediterranean, East Asian, Southeast Asian, Indian subcontinent, Ashkenazi, and Ghanaian populations, respectively [18,21,22,23,24,25].

In our current investigation, we have identified the p.Arg68Ter-*CLDN14* mutation responsible for ARNSHL in a consanguineous Syrian family. Additionally, we conducted a comprehensive review of prior *CLDN14* mutations and their corresponding phenotypes. Furthermore, we carried out a comparative analysis between the *CLDN14* and *GJB2* genes, aiming to elucidate potential factors contributing to the higher mutability of *GJB2* when compared to *CLDN14*.

## 2. Materials and Methods

### 2.1. Study Family

A Syrian consanguineous family with four affected children was recruited, and genomic DNA was isolated from saliva samples using the Oragene-DNA (OG-500) Kit (DNA Genotek, Stittsville, ON, Canada). To ensure confidentiality, the DNA samples were appropriately labeled with codes. Additionally, 100 individuals with deafness and 80 hearing participants were incorporated in this study. The parents of affected individuals provided written informed consent after audiological and clinical assessments. Moreover, the Ethics Committee at the University of Sharjah approved all the experimental methods used in this study.

### 2.2. GJB2 Screening and Whole Exome Sequencing

Individuals with the hearing loss underwent screening for *GJB2* mutations through Sanger sequencing and were analyzed using whole exome sequencing (WES) as previously described [26]. Finally, the potential functional impact of the identified variants was estimated using a variety of bioinformatics tools including Variant Effect Predictor (VEP), Mutation Taster, VarSome, PROVEAN, PolyPhen-2, SIFT, and Human Splicing Finder.

### 2.3. CLDN14 Mutational Screening

To confirm the WES results and the cosegregation of the c.202C>T (p.Arg68Ter) nonsense variant with deafness in the affected family, Sanger sequencing of the *CLDN14* coding exon was performed as described previously [12]. To summarize, after PCR amplification using CLDN14-F: ACCACCATCCTGCCGCACTG and CLDN14-R: TGTTTGCAGTGGTCGTGGTG primers, the products were purified and underwent cycle sequencing. The resulting sequences were then aligned with the publicly available sequence of the *CLDN14* gene (NM_144492).

### 2.4. In Silico Analysis

In order to ascertain the characteristics of *GJB2* and *CLDN14* genes, the following databases were used: QGRS Mapper “https://bioinformatics.ramapo.edu/QGRS/index.php” (accessed on 1 February 2024) for determining putative quadruplex forming G-rich sequences (QGRS) in nucleotide sequences, VectorBuilder “https://en.vectorbuilder.com/” (accessed on 1 February 2024) for the purpose of calculating the GC content, and GnomeAD “https://gnomad.broadinstitute.org/” (accessed on 1 February 2024) for determining the reported variants classified as pathogenic and likely pathogenic in both genes.

## 3. Results

### 3.1. Molecular Analysis

**Figure 1:**
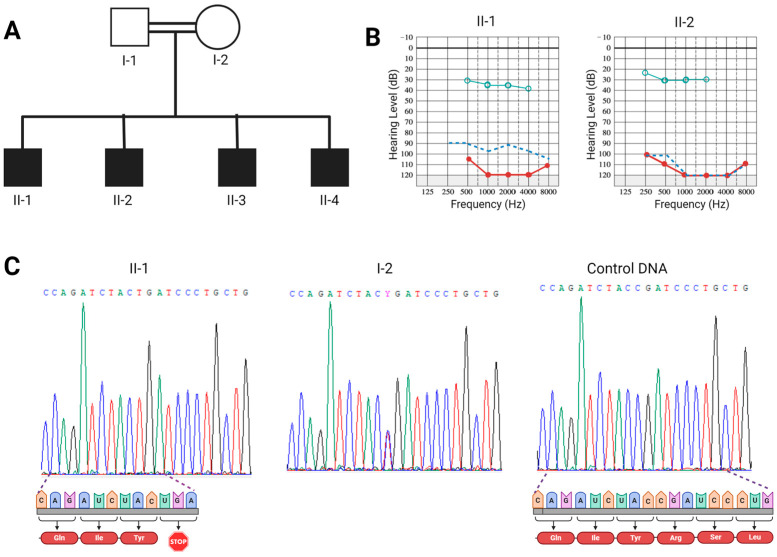
**Pedigree, Audiograms, and Electropherograms.** (**A**): Pedigree of the studied consanguineous family. (**B**) Audiograms displaying hearing threshold tests in affected individuals II-1 (16 years old) and II-2 (11 years old). The audiograms depict the right ear (in red) before cochlear implantation, as well as the left ear (in blue) and the right ear after cochlear implantation (in cyan). (**C**) Electropherograms showcasing the c.202C>T variant as homozygous in the affected individual (II-1), heterozygous in his non-deaf mother (I-2), and its absence in a control individual. Protein-level impacts of the mutant and normal alleles are indicated below the affected and normal electropherograms, respectively. Created with BioRender.com.

A consanguineous Syrian family with four affected deaf children was recruited in this study (Figure 1A). Analysis of the family pedigree suggested that the inheritance pattern is likely autosomal recessive; moreover, clinical investigation did not reveal any other abnormality and confirmed the profound hearing loss phenotype in all siblings. All affected children received cochlear implants in their right ears, resulting in an enhancement of sound field threshold levels. These levels shifted from profound hearing loss (100 or more decibels) to a mild hearing impairment (25–40 decibels) (Figure 1B). Sanger sequencing of the *GJB2* gene, the most common gene related to deafness, showed an absence of mutations in the affected individuals. Thus, we undertook whole exome sequencing for the affected individual I-1 to determine the causative mutation. Our investigation unveiled a total of 32,808 DNA variations, of which 11,726 were found to be homozygous. By choosing unknown variants or those with a frequency of less than 0.01 in the G1000 and gnomAD databases, a total of 49 DNA variations in 45 genes were obtained. After considering genes responsible for hearing loss, we narrowed down the list of candidate variants to a lone known variant located within the *CLDN14* gene. It consists of a transition C to T (c.202C>T) that substitutes the residue Arginine 68 by a stop codon (p.Arg68Ter). To validate this finding and confirm the segregation of this nonsense variant with deafness in the affected family, Sanger sequencing was carried out for the affected children and their parents. Our results revealed that all affected individuals were homozygous for this nonsense variant, whereas their parents were heterozygous (Figure 1C). This observation thereby confirms the co-segregation of c.202C>T with deafness in the investigated family. Additionally, screening for this mutation in 100 unrelated individuals with deafness and 80 hearing participants revealed that it was absent in all cases.

### 3.2. CLDN14 Mutations and Phenotypic Variations

**Figure 2:**
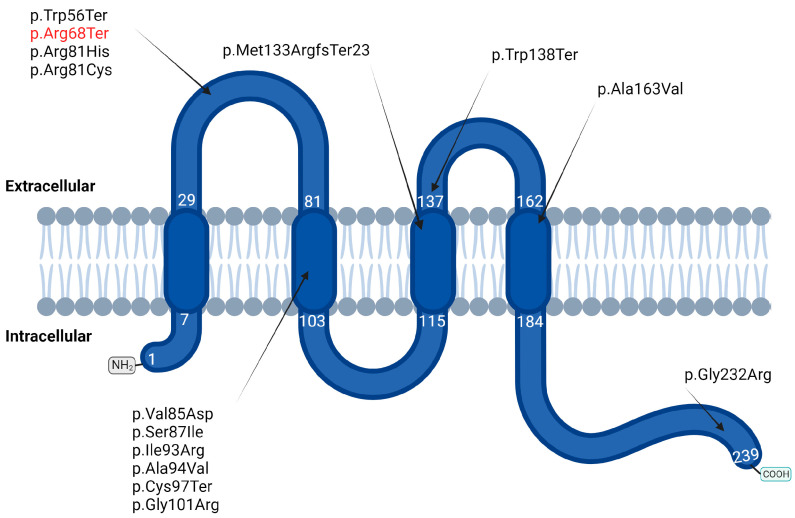
**Schematic representation of Claudin-14 mapping the locations of pathogenic mutations, with the identified mutation in red** (numeric annotations indicate amino acid positions defining extracellular and intracellular loops). Created with BioRender.com.

An overview of reported *CLDN14* mutations shows that, so far, 14 mutations are associated with ARNSHL (Table 1). The majority of these mutations occur in the second transmembrane domain, and most of them are associated with a severe to profound phenotype (p.Arg81His, p.Ser87Ile, and p.Gly232Arg), with the notable exception of p.Ala94Val and p.Val85Asp which have been associated with moderate to severe hearing loss (Figure 2). It is worth mentioning that five mutations are truncated with four nonsense variants (p.Trp56Ter, p.Arg68Ter, p.Cys97Ter and p.Trp138Ter) and one frameshift (p.Met133ArgfsTer23). Phenotypic details are available for p.Trp138Ter and p.Met133ArgfsTer23, showing a hearing loss phenotype of moderate to profound severity in both cases (Table 1). Although the impact of *CLDN14* mutations tends to increase in severity at higher frequencies, the less severe phenotype was observed at higher frequencies for two mutations that involve the replacement of an Alanine residue by a Valine amino acid (p.Ala94Val and p.Ala163Val).

Finally, our review analysis revealed the existence of four variants reported as founder mutations across various populations. These mutations, p.Met133ArgfsX23 and p.Val85Asp, were both identified as founder variants within the Pakistani population, whereas p.Trp138Ter was identified as a founder in the Yemeni population, and p.Ala163Val was reported in the Newfoundland population.

### 3.3. Comparative Analysis of CLDN14 and GJB2 Genes

Since *CLDN14* and *GJB2* genes both possess coding regions of roughly equivalent sizes, albeit with distinct mutational rates, we tried to identify potential parameters that might explain the low frequency and number of mutations in *CLDN14* compared to *GJB2*. In fact, using the gnomAD database, a brief review of pathogenic and/or likely pathogenic variants in these two genes demonstrated the presence of 74 variants in *GJB2*, while the *CLDN14* gene showed only 4 (Table 2). To provide an explanatory insight, we investigated their chromosomal locations, their nucleotide sequences, and the number of putative QGRS. Our analysis showed that the *GJB2* gene is very close to the centromeric region with a distance of approximately 1.29 Mb from the centromere, while the *CLDN14* gene was located at a distance of 23.46 Mb from the centromeric region. The investigation into the coding regions of these two genes revealed that the *GJB2* nucleotide sequence has a significant cytosine content, accounting for 37.5% (A (17.5% 126)|C (37.5% 270)|G (26.81% 193)|T (18.19% 131)). In contrast, the cytosine content of the *CLDN14* gene amounts to 25.55% (A (23.2% 158)|C (25.55% 174)|G (26.58% 181)|T (24, 67% 168)), which represents a perceptible difference between the two. Finally, the investigation of both nucleotide sequences unveiled the presence of 17 potential QGRS within the *GJB2* gene, while the *CLDN14* gene revealed a more limited number, with only 4 identified QGRS.

## 4. Discussion

In the current study, we investigated a Syrian family with ARNSHL. Affected individuals showed profound hearing loss. The analysis of *GJB2*, which is the primary gene contributing to ARNSHL [27], revealed the absence of pathogenic variants. To identify the responsible DNA variation, WES, which represents an effective method in identifying HL causative mutations [28], was performed for the affected individuals. This approach unveiled the presence of the nonsense DNA variant c.202C>T (p.Arg68Ter) in the *CLDN14* gene, which segregates with HL in the studied family. This mutation was previously identified in a simplex Chinese family, but details regarding its segregation and associated phenotype were not provided [17].

Claudin 14, belonging to the claudin protein family, is an essential membrane protein that plays a key role in regulating calcium levels and maintaining ion balance. Various variants of the *CLDN14* gene have been linked to hearing loss and the formation of kidney stones [29]. In the knockout mouse model (*Cldn14−/−*), a degeneration of outer hair cells starts during the first postnatal week, followed by the deterioration of the inner hair cells in the subsequent week [6]. The deterioration of hair cells likely results from the exposure of the hair cell’s basolateral areas to a potassium-rich endolymph, which is facilitated by a defect in the ion barrier impairment due to the absence of the tight junction protein Claudin 14 [6]. To date, 14 pathogenic mutations have been associated with ARNSHL in humans, of which 9 are missense mutations, 3 are nonsense mutations and 1 is a frameshift deletion (Table 1). The phenotype associated with 9 of these variants has been reported, showing a greater degree of hearing loss severity at higher frequencies compared to lower frequencies, which correlates with the degeneration pattern reported in the knockout mouse where the apical region showed more pronounced hair cell degeneration and a more significant loss of presynaptic ribbons compared to the basal region [30]. In this report, it is noteworthy that all affected siblings display profound hearing loss across all frequencies. This stands in contrast to previous mutations reported and the knockout mouse model, where a clear severity gradient was observed. This variation could potentially be attributed to the complete absence or significantly reduced size of the polypeptide resulting from the c.202C>T (p.Arg68Ter) mutation, which lacks both extracellular loops (ECL1 and ECL2). These domains are very important in the regular function of claudins, as ECL1 is vital for controlling the tightness of paracellular junctions and selective ion permeability, whereas ECL2 can lead to a reduction in the width of the paracellular cleft [31].

Moreover, the *Cldn14* knockout mice have shown the loss of ribbon synapses in inner hair cells and regression of auditory nerve fibers [30]. Given the important role of the auditory nerve in cochlear-implant-based auditory rehabilitation [32], Claußen et al. proposed exploring the performance of cochlear implantation in Cldn14−/− mice in the future [30]. In the present study, the four deaf children with the c.202C>T (p.Arg68Ter) nonsense variant do not appear to exhibit defects in their auditory nerves, as their cochlear implants have successfully improved their hearing levels. This situation was also reported in a prior investigation, where a cochlear implant was carried out on a patient with the missense mutation p.Arg81Cys, leading to an increase in threshold levels from 77.5 decibels to 37.5 [16].

Finally, the comparison between *CLDN14* and *GJB2* genes allowed us to identify some potential factors that can explain the low rate of mutations in the *CLDN14* gene compared to *GJB2*. In fact, it appears that chromosomal location seems to play a role in this difference. Many studies have demonstrated that centromeric regions, particularly the heterochromatin ones, tend to undergo late replication [33]. This pattern of late replication leads to a two-fold increase in transition DNA variations and a six-fold rise in transversion DNA variations, in comparison to loci that replicate early [34].

Additionally, the nucleotide composition can be a contributing factor to the lower mutation rate observed in the *CLDN14* gene when compared to *GJB2*. The *CLDN14* gene has a C content of 25.55%, whereas the GJB2 gene has a C content of 37.5%. This elevated C content in the *GJB2* gene may explain its increased mutation rate as previous studies have showed that Cytosines exhibit a germline mutation rate roughly tenfold greater than that observed in the other nucleotides [35]. Finally, the analysis of both nucleotide sequences showed 17 potential QGRS within the *GJB2* gene versus 4 in the *CLDN14* gene. The high number of QGRS in the *GJB2* gene can be also a contributor to the high mutational rate observed in this gene compared to the *CLDN14* one, as many studies showed an association between QGRS and high mutation rates [36].

## Figures and Tables

**Table 1:** Summary of *CLDN14* mutations and their associated phenotypes.

*DNA Mutation*	*Protein Mutation*	*Phenotype*	*References*
c.167G>A	p.Trp56Ter	N/A	[10]
c.241C>T	p.Arg81Cys	M to P	[16]
c.242G>A	p.Arg81His	S to P	[10]
c.254T>A	p.Val85Asp	S to P	[10]
c.259_260TC>AT	p.Ser87Ile	S to P	[7]
c.278 T > G	p.Ile93Arg	N/A	[9]
c.281C>T	p.Ala94Val	M to S	[7]
c.291C>A	p.Cys97Ter	N/A	[11]
c.301G>A	p.Gly101Arg	N/A	[15]
c.398delT	p.Met133ArgfsTer23	M to P	[7,10]
c.414G>A	p.Trp138Ter	M to P	[12]
c.488C>T	p.Ala163Val	Mi to S	[14]
c.694G>A	p.Gly232Arg	S to P	[10]

N/A: Not available; Mi: mild; M: moderate; S: severe; P: profound.

**Table 2:** Variants classified as pathogenic and/or likely pathogenic in GnomAD.

*GJB2* Variants	*CLDN14* Variants
c.675A>T; c.672G>A; c.632G>A; c.606C>T; c.597T>C; c.583A>G; c.563A>G; c.550C>T; c.546G>A; c.523_533del; c.500T>C; c.490C>T; c.477C>T; c.456C>T; c.444C>T; c.438C>T; c.427C>T; c.407dup; c.396G>A; c.393C>G; c.370C>T; c.358_360del; c.321C>T; c.299_300del; c.299A>T; c.290dup; c.269dup; c.267C>G; c.264G>A; c.264G>C; c.258G>A; c.258G>T; c.235del; c.231G>A; c.229T>C; c.228A>G; c.227T>C; c.225G>T; c.224G>A; c.208C>G; c.203A>G; c.195C>T; c.186C>T; c.177C>T; c.169C>T; c.167del; c.139G>T; c.132G>A; c.128T>C; c.119C>A; c.110T>C; c.109G>A; c.108C>G; c.101T>C; c.95G>A; c.94C>T; c.78C>T; c.71G>A; c.56G>T; c.41dup; c.41A>G; c.40A>G; c.39G>A; c.35del; c.34G>T; c.33G>T; c.30G>C; c.28del; c.21G>A; c.15G>A; c.2T>C; c.1A>G; c.-22-2A>C; c.-57G>T.	c.414G>A; c.254T>A; c.242G>A; c.167G>A.

## Data Availability

All research data are available in the submitted article.

## References

[1] Morton N.E. (1991). Genetic epidemiology of hearing impairment. Ann. N. Y. Acad. Sci..

[2] Smith R.J., Bale J.F., White K.R. (2005). Sensorineural hearing loss in children. Lancet.

[3] Wilcox E.R., Burton Q.L., Naz S., Riazuddin S., Smith T.N., Ploplis B., Belyantseva I., Ben-Yosef T., Liburd N.A., Morell R.J. (2001). Mutations in the gene encoding tight junction claudin-14 cause autosomal recessive deafness DFNB29. Cell.

[4] Riazuddin S., Ahmed Z.M., Fanning A.S., Lagziel A., Kitajiri S., Ramzan K., Khan S.N., Chattaraj P., Friedman P.L., Anderson J.M. (2006). Tricellulin is a tight-junction protein necessary for hearing. Am. J. Hum. Genet..

[5] Kitajiri S., Furuse M., Morita K., Saishin-Kiuchi Y., Kido H., Ito J., Tsukita S. (2004). Expression patterns of claudins, tight junction adhesion molecules, in the inner ear. Hear Res..

[6] Ben-Yosef T., Belyantseva I.A., Saunders T.L., Hughes E.D., Kawamoto K., Van Itallie C.M., Beyer L.A., Halsey K., Gardner D.J., Wilcox E.R. (2003). Claudin 14 knockout mice, a model for autosomal recessive deafness DFNB29, are deaf due to cochlear hair cell degeneration. Hum. Mol. Genet..

[7] Bashir Z., Latief N., Belyantseva I.A., Iqbal F., Riazuddin S.A., Khan S.N., Friedman T.B., Riazuddin S., Riazuddin S. (2013). Phenotypic variability of CLDN14 mutations causing DFNB29 hearing loss in the Pakistani population. J. Hum. Genet..

[8] Charif M., Boulouiz R., Bakhechane A., Benrahma H., Nahili H., Eloualid A., Rouba H., Kandil M., Abidi O., Lenaers G. (2013). Genetic and molecular analysis of the CLDN14 gene in Moroccan family with non-syndromic hearing loss. Indian J. Hum. Genet..

[9] Elsayed O., Al-Shamsi A. (2022). Mutation spectrum of non-syndromic hearing loss in the UAE, a retrospective cohort study and literature review. Mol. Genet. Genom. Med..

[10] Lee K., Ansar M., Andrade P.B., Khan B., Santos-Cortez R.L.P., Ahmad W., Leal S.M. (2012). Novel CLDN14 mutations in Pakistani families with autosomal recessive non-syndromic hearing loss. Am. J. Med. Genet. A.

[11] Manzoli G.N., Bademci G., Acosta A.X., Félix T.M., Cengiz F.B., Foster J., Da Silva D.S.D., Menendez I., Sanchez-Pena I., Tekin D. (2016). Targeted Resequencing of Deafness Genes Reveals a Founder MYO15A Variant in Northeastern Brazil. Ann. Hum. Genet..

[12] Mohamed W.K.E., Mahfood M., Al Mutery A., Abdallah S.H., Tlili A. (2019). A Novel Nonsense Mutation (c.414G>A; p.Trp138*) in CLDN14 Causes Hearing Loss in Yemeni Families: A Case Report. Front. Genet..

[13] Pandey N., Rashid T., Jalvi R., Sharma M., Rangasayee R., Andrabi K.I., Anand A. (2017). Mutations in OTOF, CLDN14 & SLC26A4 genes as major causes of hearing impairment in Dhadkai village, Jammu & Kashmir, India. Indian J. Med. Res..

[14] Pater J.A., Benteau T., Griffin A., Penney C., Stanton S.G., Predham S., Kielley B., Squires J., Zhou J., Li Q. (2017). A common variant in CLDN14 causes precipitous, prelingual sensorineural hearing loss in multiple families due to founder effect. Hum. Genet..

[15] Wattenhofer M., Reymond A., Falciola V., Charollais A., Caille D., Borel C., Lyle R., Estivill X., Petersen M.B., Meda P. (2005). Different mechanisms preclude mutant CLDN14 proteins from forming tight junctions in vitro. Hum. Mutat..

[16] Kitano T., Kitajiri S., Nishio S., Usami S. (2019). Detailed Clinical Features of Deafness Caused by a Claudin-14 Variant. Int. J. Mol. Sci..

[17] Li Y., Su J., Zhang J., Pei J., Li D., Zhang Y., Li J., Chen M., Zhu B. (2021). Targeted next-generation sequencing of deaf patients from Southwestern China. Mol. Genet. Genom. Med..

[18] Kelsell D.P., Dunlop J., Stevens H.P., Lench N.J., Liang J.N., Parry G., Mueller R.F., Leigh I.M. (1997). Connexin 26 mutations in hereditary non-syndromic sensorineural deafness. Nature.

[19] Beach R., Abitbol J.M., Allman B.L., Esseltine J.L., Shao Q., Laird D.W. (2020). GJB2 Mutations Linked to Hearing Loss Exhibit Differential Trafficking and Functional Defects as Revealed in Cochlear-Relevant Cells. Front. Cell Dev. Biol..

[20] Wingard J.C., Zhao H.B. (2015). Cellular and Deafness Mechanisms Underlying Connexin Mutation-Induced Hearing Loss—A Common Hereditary Deafness. Front. Cell. Neurosci..

[21] Brobby G.W., Muller-Myhsok B., Horstmann R.D. (1998). Connexin 26 R143W mutation associated with recessive nonsyndromic sensorineural deafness in Africa. N. Engl. J. Med..

[22] Zelante L., Gasparini P., Estivill X., Melchionda S., D’Agruma L., Govea N., Mila M., Monica M.D., Lutfi J., Shohat M. (1997). Connexin26 mutations associated with the most common form of non-syndromic neurosensory autosomal recessive deafness (DFNB1) in Mediterraneans. Hum. Mol. Genet..

[23] Kelley P.M., Harris D.J., Comer B.C., Askew J.W., Fowler T., Smith S.D., Kimberling W.J. (1998). Novel mutations in the connexin 26 gene (GJB2) that cause autosomal recessive (DFNB1) hearing loss. Am. J. Hum. Genet..

[24] Fuse Y., Doi K., Hasegawa T., Sugii A., Hibino H., Kubo T. (1999). Three novel connexin26 gene mutations in autosomal recessive non-syndromic deafness. Neuroreport.

[25] Lucotte G. (2007). High prevalences of carriers of the 35delG mutation of connexin 26 in the Mediterranean area. Int. J. Pediatr. Otorhinolaryngol..

[26] Mahfood M., Chouchen J., Kamal Eddine Ahmad Mohamed W., Al Mutery A., Harati R., Tlili A. (2021). Whole exome sequencing, in silico and functional studies confirm the association of the GJB2 mutation p.Cys169Tyr with deafness and suggest a role for the TMEM59 gene in the hearing process. Saudi J. Biol. Sci..

[27] Al-Achkar W., Moassass F., Al-Halabi B., Al-Ablog A. (2011). Mutations of the Connexin 26 gene in families with non-syndromic hearing loss. Mol. Med. Rep..

[28] Atik T., Bademci G., Diaz-Horta O., Blanton S.H., Tekin M. (2015). Whole-exome sequencing and its impact in hereditary hearing loss. Genet. Res..

[29] Ullah I., Murtaza K., Ammara H., Misbah, Bhinder M.A., Riaz A., Shehzad W., Zahoor M.Y. (2022). Association study of CLDN14 variations in patients with kidney stones. Open Life Sci..

[30] Claußen M., Schulze J., Nothwang H.G. (2020). Loss of inner hair cell ribbon synapses and auditory nerve fiber regression in Cldn14 knockout mice. Hear Res..

[31] Krause G., Winkler L., Piehl C., Blasig I., Piontek J., Müller S.L. (2009). Structure and function of extracellular claudin domains. Ann. N. Y. Acad. Sci..

[32] Maxwell A.P., Mason S.M., O’Donoghue G.M. (1999). Cochlear nerve aplasia: Its importance in cochlear implantation. Am. J. Otol..

[33] Nesta A.V., Tafur D., Beck C.R. (2021). Hotspots of Human Mutation. Trends Genet..

[34] Kim S., Dubey D.D., Huberman J.A. (2003). Early-replicating heterochromatin. Genes Dev..

[35] Nachman M.W., Crowell S.L. (2000). Estimate of the mutation rate per nucleotide in humans. Genetics.

[36] Guiblet W.M., Cremona M.A., Harris R.S., Chen D., Eckert K.A., Chiaromonte F., Huang Y., Makova K.D. (2021). Non-B DNA: A major contributor to small- and large-scale variation in nucleotide substitution frequencies across the genome. Nucleic Acids Res..

